# Changepoint Detection in Heart Rate Variability Indices in Older Patients Without Cancer at End of Life Using Ballistocardiography Signals: Preliminary Retrospective Study

**DOI:** 10.2196/53453

**Published:** 2024-02-12

**Authors:** Naotake Yanagisawa, Yuji Nishizaki, Bingwei Yao, Jianting Zhang, Takatoshi Kasai

**Affiliations:** 1 Medical Technology Innovation Center Juntendo University Tokyo Japan; 2 Division of Medical Education Juntendo University School of Medicine Tokyo Japan; 3 E3 Enterprise Tokyo Japan; 4 Zhejiang Huiyang Technology Huzhou China; 5 Department of Cardiovascular Biology and Medicine Juntendo University Graduate School of Medicine Tokyo Japan

**Keywords:** ballistocardiography, BCG, nonnvasive monitoring, heart rate variability, end-of-life care, prognosis prediction

## Abstract

**Background:**

In an aging society such as Japan, where the number of older people continues to increase, providing in-hospital end-of-life care for all deaths, and end-of-life care outside of hospitals, such as at home or in nursing homes, will be difficult. In end-of-life care, monitoring patients is important to understand their condition and predict survival time; this information gives family members and caregivers time to prepare for the end of life. However, with no clear indicators, health care providers must subjectively decide if an older patient is in the end-of-life stage, considering factors such as condition changes and decreased food intake. This complicates decisions for family members, especially during home-based care.

**Objective:**

The purpose of this preliminary retrospective study was to determine whether and how changes in heart rate variability (HRV) indices estimated from ballistocardiography (BCG) occur before the date of death in terminally ill older patients, and ultimately to predict the date of death from the changepoint.

**Methods:**

This retrospective pilot study assessed the medical records of 15 older patients admitted to a special nursing home between August 2019 and December 2021. Patient characteristics and time-domain HRV indices such as the average normal-to-normal (ANN) interval, SD of the normal-to-normal (SDNN) interval, and root mean square of successive differences (RMSSD) from at least 2 months before the date of death were collected. Overall trends of indices were examined by drawing a restricted cubic spline curve. A repeated measures ANOVA was performed to evaluate changes in the indices over the observation period. To explore more detailed changes in HRV, a piecewise regression analysis was conducted to estimate the changepoint of HRV indices.

**Results:**

The 15 patients included 8 men and 7 women with a median age of 93 (IQR 91-96) years. The cubic spline curve showed a gradual decline of indices from approximately 30 days before the patients’ deaths. The repeated measures ANOVA showed that when compared with 8 weeks before death, the ratio of the geometric mean of ANN (0.90, 95% CI 0.84-0.98; *P*=.005) and RMSSD (0.83, 95% CI 0.70-0.99; *P*=.03) began to decrease 3 weeks before death. The piecewise regression analysis estimated the changepoints for ANN, SDNN, and RMSSD at –34.5 (95% CI –42.5 to –26.5; *P*<.001), –33.0 (95% CI –40.9 to –25.1; *P*<.001), and –35.0 (95% CI –42.3 to –27.7; *P*<.001) days, respectively, before death.

**Conclusions:**

This preliminary study identified the changepoint of HRV indices before death in older patients at end of life. Although few data were examined, our findings indicated that HRV indices from BCG can be useful for monitoring and predicting survival time in older patients at end of life. The study and results suggest the potential for more objective and accurate prognostic tools in predicting end-of-life outcomes.

## Introduction

### Background

The population of Japan was estimated to be 125 million as of October 1, 2020, and has been declining for 11 consecutive years since 2011 [1]. Meanwhile, the aging rate is increasing, and the number of people aged 65 years and older is estimated at 36 million, accounting for 28.8% of the total population. As the total population declines further, this percentage is expected to continue to increase, reaching approximately 33.3% in 2036 and 38.4% in 2065 [2].

The number of deaths has also increased over the years, exceeding 1 million in 2003 and 1.37 million in 2020. Malignant neoplasms are the leading cause of death, accounting for 27.6% of all deaths, followed by heart disease (15%) and senility (9.6%) [3]. Notably, in 2018, deaths due to senility surpassed cerebrovascular disease and became the third leading cause of death [4], possibly due to the growing number of people aged 90 years and older. The increasing number of deaths among older people creates difficulty in caring for all deaths in hospitals. Therefore, it is expected that end-of-life care outside hospitals will increasingly be provided in nursing homes and at home. In end-of-life care, it is important to monitor patients to understand their condition and predict survival time; this information helps patients anticipate what is to come and gives family members and caregivers time to prepare for the end of life [5,6].

However, this process may be very difficult under conditions when resources are limited, such that medical staff may not be available immediately at home. Furthermore, the physical, spiritual, and psychosocial conditions of older patients at end of life vary [7]. Unnecessary monitoring is also burdensome to patients and should be discouraged. To reduce the burden on patients and families, noninvasive and nonintrusive monitoring systems have been increasingly important, and several studies have demonstrated their usefulness in palliative care [8-10]. While tools to predict the survival of patients with terminal cancer have been developed and many studies have shown their usefulness [11-13], the reliability of these tools in predicting survival time for end-of-life patients without cancer has been questionable, and the tools are considered difficult to use [14,15]. Therefore, we focused on continuous and unobtrusive monitoring of patients using heart rate variability (HRV) indices estimated from ballistocardiography (BCG) obtained from a sheet-type device as a means of monitoring their condition. BCG is a measurement of the vibration signals generated by the ejection of the blood at each cardiac cycle [16], which has been used frequently in recent years to acquire biological signals [17-20]. The advantages of this method are that BCG signals can be obtained by placing a device under a patient’s mattress, making long-term, around-the-clock monitoring possible without attaching electrodes to the patient. The device could also reduce the burden on nurses by allowing them to monitor patients remotely. For example, many recent applied studies have been conducted using BCG to detect hypertension and apnea using HRV indices estimated from BCG signals [21-26]. HRV is a measure of the variation in time between heartbeats, usually recorded by an electrocardiogram (ECG). This variation is related to the autonomic nervous system and reflects a person’s health status [27]. A decrease in HRV generally results in a higher mortality rate [28,29] in patients with myocardial infarction, and HRV has also been demonstrated to be associated with a variety of diseases [30-33]. If the prognosis, including the patient’s survival, can be accurately estimated by HRV indices through noninvasive and noncontact monitoring, this would be beneficial for end-of-life patients, as well as their clinicians, caregivers, and family members, to plan future care and prepare for the end of life.

### Objectives

The aim of this preliminary retrospective study was to investigate whether there is a changepoint in HRV indices obtained from BCG signals prior to death to predict the survival time in older patients without cancer at end of life.

## Methods

### Study Design and Patients

This was a single-center, retrospective pilot study in which all medical records of end-of-life patients (median age 93, IQR 91-96 years) were extracted from one nursing hospital in China. The study included data from 15 patients who were admitted to the facility and died between August 1, 2019, and December 31, 2021. All patients were without cancer. Patients with implanted pacemakers and patients with chronic atrial fibrillation or frequent extrasystoles were excluded. In addition, patients with less than 2 months of data from the date of death were excluded.

### Measurements

Age and sex were collected as patient characteristics, and the following classical time-domain HRV indices were obtained from a medical database and used for analyses: average normal-to-normal (ANN) interval for each 5-minute segment of HRV recording; SD of all normal-to-normal (SDNN) intervals; and root mean square of successive differences (RMSSD). These HRV indices were collected up to 2 months before the patients’ deaths and used for data analyses.

### BCG Sensing Device

**Figure 1:**
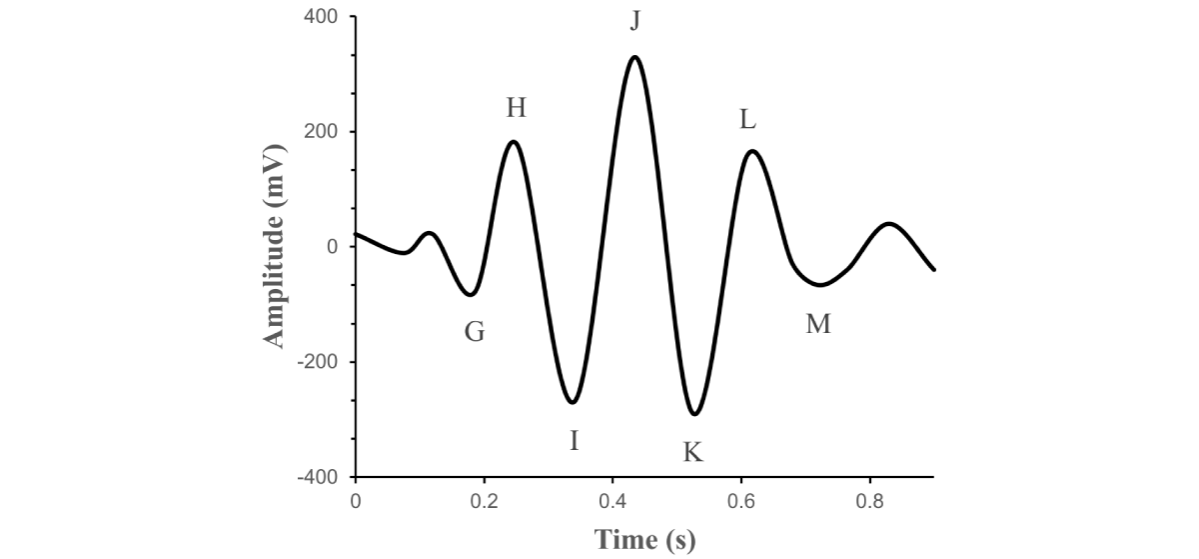
Typical wave form of ballistocardiography signal.

The BCG device used in this study is made by Zhejiang Huiyang Technology Co, Ltd; it is a sheet-type device equipped with a highly sensitive motion detection sensor that is placed under pillows or mattresses to continuously measure the patient’s body movements. In addition to HRV indices, this device can measure arousal, sleep, respiratory rate, and bed-withdrawal time. HRV parameters were processed from continuously recorded raw BCG signals at 133 Hz. The BCG signal was preprocessed through filtering and detection of the BCG wave (the J-wave) based on proprietary algorithms. The typical wave form of BCG signals is shown in Figure 1. After detection of J-J wave intervals, ectopic intervals were excluded in time series and formed normal-to-normal intervals. These intervals were used as an alternative to the RR interval (RRI) obtained from ordinary ECGs.

### Statistical Analysis

Data are summarized as mean (SD) for continuous variables if the data followed normal distribution and as median (IQR) otherwise. Categorical variables are expressed as numbers and percentages. The time-course changes of HRV indices were examined by drawing a spline chart with a restricted cubic spline model and 3 internal knots fitting each HRV index as a function of time. A repeated measures ANOVA model was conducted to evaluate the within-subject effect, that is, the average of an individual trend of HRV indices over the period for which data were obtained. Since multiple analyses were performed with the reference and the values at each time point, multiplicity was adjusted using the Dunnett-Hsu method. Before analysis, data were natural-log transformed to reduce the right-skewness of the original data, and then daily or weekly averages were calculated for each patient. After analysis, estimates were back-transformed. The results at each time point represent the ratio of the geometric mean of values to reference values. To explore the location of the changepoint of HRV indices over the observation period, a piecewise regression model was used. The regression coefficients were back-transformed; the regression coefficients represent the fold-change for every 10-unit change in the independent variables. The statistical 2-sided significance level was set at .05, and *P*<.05 was considered statistically significant. All statistical analyses were conducted using SAS (version 9.4; SAS Institute).

### Ethical Considerations

The study was conducted in accordance with the Declaration of Helsinki (revised in 2013) and the Ethical Guidelines for Medical and Health Research Involving Human Subjects in Japan (revised in 2017). The study received approval from the Research Ethics Committee, Faculty of Medicine, Juntendo University (M19-0287). The committee waived the requirement for informed consent for this retrospective study, as it involved the analysis of existing data. All study data were anonymized, and any identifying information was removed to ensure the complete confidentiality of patients. Only authorized research personnel had access to the collected data, and any publication or presentation of the results strictly maintained the anonymity of the study. Patients were not offered any compensation for their participation in this study.

## Results

### Patient Characteristics

A summary of patient demographic characteristics is presented in Table 1. The data were obtained from 15 patients aged 90 to 100 years, including 8 men and 7 women. The median age for the entire group was 93 (IQR 91-96) years, and by sex, the median age was 94 (IQR 90-97) years for women and 93 (IQR 91.5-94.5) years for men. The other underlying cardiac diseases of the patients were not a concern; however, only cases with no change in medication during the period were included, resulting in a final study total of 15 patients.

**Table 1:** Characteristics of end-of-life older patients (N=15).

Characteristics		Values
Age (years), median (IQR)		93 (91-96)
**Sex, n (%)**		
	Female	7 (47)
	Male	8 (53)

### Time-Course Changes of HRV Indices

**Figure 2:**
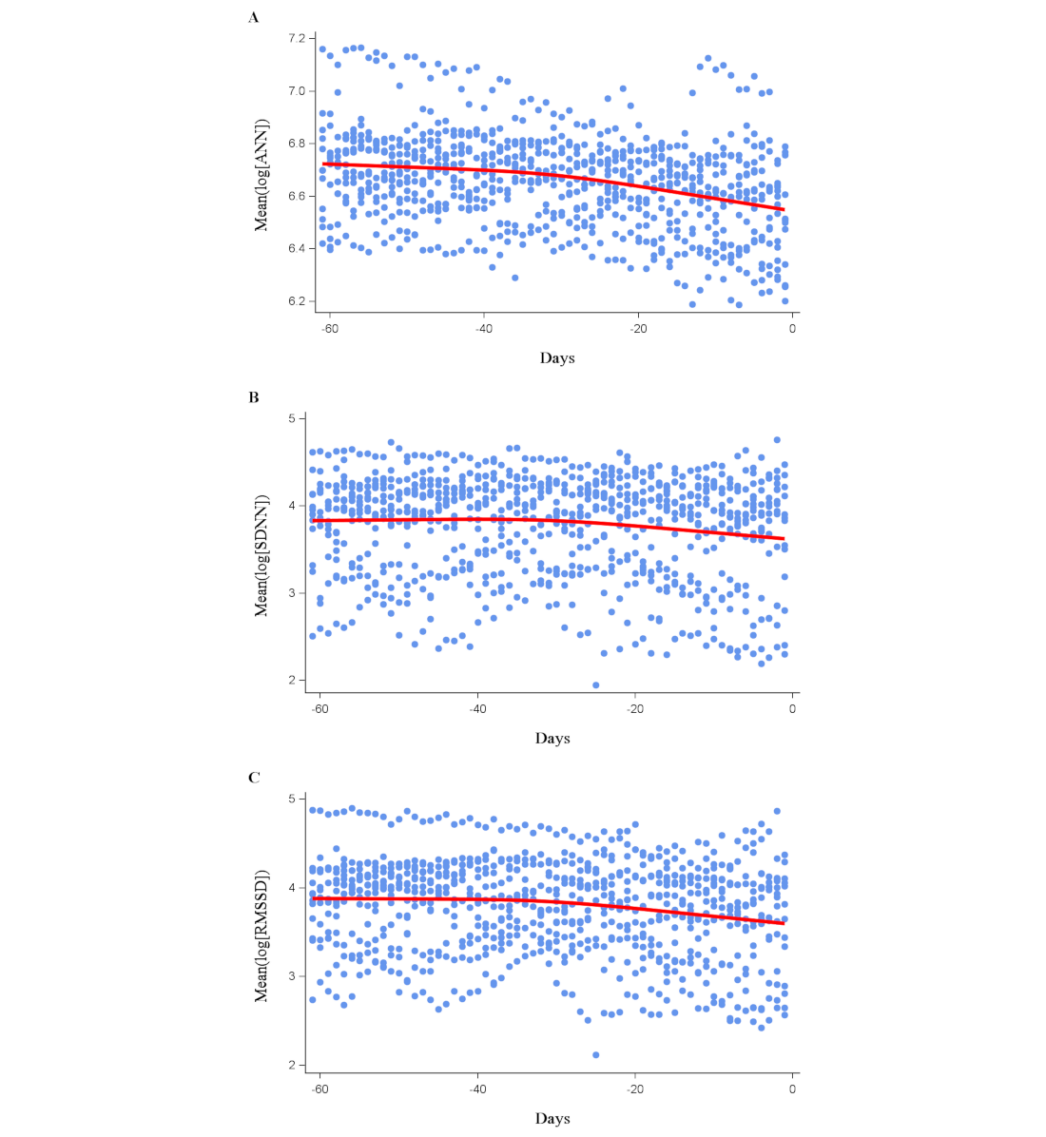
Spline curve chart of time-course changes in heart rate variability indices for (A) average normal-to-normal (ANN), (B) SD of all normal-to-normal (SDNN), and (C) root mean square of successive differences (RMSSD).

Time-course changes in HRV indices were plotted by spline charts (Figure 2). The x-axis in this figure represents the date of the event as 0 and proceeds backwards from there. As shown in Figure 2, the value for ANN gradually decreased from approximately –60 days to approximately –30 days, but the degree of the trend slightly increased after approximately –30 days until the patients’ deaths. A similar trend was observed in RMSSD and SDNN; however, for SDNN, the values seemed to be constant between –60 days to approximately –30 days. After –30 days, a decreasing trend was observed.

### Repeated Measures ANOVA

The results of the repeated measures ANOVA are shown in Table 2. In this analysis, the average of the log-transformed data at 8 weeks before the patients’ deaths was used as a reference and compared to each averaged log-transformed result from 7 weeks to 1 week before death. Then, the estimates were back-transformed in order to represent the results as the ratios to reference. At 3, 2, and 1 week before the patients’ deaths, there were significant decreases in ANN (3 w: 0.90, 95% CI 0.84-0.98; *P*=.005; 2 w: 0.90, 95% CI 0.84-0.98; *P*=.004; 1 w: 0.86, 95% CI 0.80-0.93; *P*<.001) and RMSSD (3 w: 0.83, 95% CI 0.70-0.99; *P*=.03; 2 w: 0.83, 95% CI 0.70-1.00; *P*=.04; 1 w: 0.78, 95% CI 0.66-0.93; *P*=.002) compared to the reference. Although not statistically significant, SDNN began to decrease from 4 weeks, and a statistically significant decrease was observed at 1 week (0.81, 95% CI 0.67-0.97; *P*=.02) compared to the reference.

**Table 2:** Repeated measures ANOVA for heart rate variability indices. The values represent ratios of each week’s value to the reference and their corresponding 95% CIs.

Weeks before the patients’ deaths	Average normal-to-normal interval (95% CI)	*P* value^a^	SD of all normal-to-normal intervals (95% CI)	*P* value^a^	Root mean square of successive differences (95% CI)	*P* value^a^
8	Reference	>.99	Reference	>.99	Reference	>.99
7	1.00 (0.93-1.08)	>.99	0.99 (0.83-1.19)	.99	0.99 (0.83-1.18)	>.99
6	1.00 (0.92-1.08)	>.99	1.00 (0.83-1.21)	>.99	0.99 (0.83-1.18)	>.99
5	0.98 (0.91-1.06)	.99	1.01 (0.84-1.22)	>.99	0.90 (0.76-1.07)	>.99
4	0.94 (0.87-1.02)	.21	0.96 (0.80-1.16)	.99	0.90 (0.76-1.07)	.43
3	0.90 (0.84-0.98)	.005	0.88 (0.73-1.05)	.26	0.83 (0.70-0.99)	.03
2	0.90 (0.84-0.98)	.004	0.85 (0.70-1.02)	.09	0.83 (0.70-1.00)	.04
1	0.86 (0.80-0.93)	<.001	0.81 (0.67-0.97)	.02	0.78 (0.66-0.93)	.002
*P* value^b^	<.001		.005		<.001	

^a^These *P* values represent the results at each time point relative to the reference, that is, the Dunnett-Hsu–adjusted *P* value.

^b^These *P* values represent the results for the change in the heart rate variability indices over the entire observation period.

### Changepoint Estimation

The changepoints in HRV indices were explored by using a piecewise regression model. Results are shown in Table 3. The changepoints for ANN, SDNN, and RMSSD were estimated as occurring at –34.5 (95% CI –42.5 to –26.5), –33.0 (95% CI –40.9 to –25.1), and –35.0 (95% CI –42.3 to –27.7) days, respectively, before the date of the patient’s death at 0. The regression coefficients of the slope for the HRV indices before the changepoint were not statistically significantly different from 0; however, after the changepoint, the coefficients indicated that HRV indices were significantly decreased by a factor of the coefficients for every 10 units of increase in days (ANN: 0.959, 95% CI 0.950-0.968; *P*<.001; SDNN: 0.930, 95% CI 0.908-0.953; *P*<.001; RMSSD: 0.925, 95% CI 0.905-0.946; *P*<.001).

**Table 3:** Changepoint analysis in heart rate variability indices.

Variables		Estimates (95% CI)	*P* value
**Average normal-to-normal interval**			
	Intercept before changepoint	793.9 (717.7 to 878.1)	<.001
	Slope^a^ before changepoint	0.993 (0.980 to 1.006)	.25
	Intercept after changepoint	705.1 (650.9 to 763.8)	<.001
	Slope^a^ after changepoint	0.959 (0.950 to 0.968)	<.001
	Changepoint (days)^b^	–34.5 (–42.5 to –26.5)	<.001
**SD of all normal-to-normal intervals**			
	Intercept before changepoint	51.8 (37.4 to 71.7)	< .001
	Slope^a^ before changepoint	1.020 (0.986 to 1.055)	.23
	Intercept after changepoint	38.2 (28.8 to 50.9)	<.001
	Slope^a^ after changepoint	0.930 (0.908 to 0.953)	<.001
	Changepoint (days)^b^	–33.0 (–40.9 to –25.1)	<.001
**Root mean square of successive differences**			
	Intercept before changepoint	51.4 (37.9 to 69.6)	< .001
	Slope^a^ before changepoint	1.011 (0.980 to 1.043)	.45
	Intercept after changepoint	37.6 (28.9 to 49.1)	<.001
	Slope^a^ after changepoint	0.925 (0.905 to 0.946)	<.001
	Changepoint (days)^b^	–35.0 (–42.3 to –27.7)	<.001

^a^The regression coefficient of slope indicates that for every 10-unit increase in days, heart rate variability indices increase or decrease by the value multiplied by the regression coefficient.

^b^Values for changepoint represent days before the date of the patient’s death at 0.

## Discussion

### Principal Findings

This preliminary retrospective study aimed to explore the changepoint of HRV indices based on estimates from BCG signals measured over a period of time in older patients at end of life who were residents in a nursing home. Our analysis, conducted by plotting a spline curve, revealed a gradual decline in HRV indices from approximately 30 days before the patients’ deaths. To assess whether this change could be detected statistically, we compared the HRV indices 8 weeks before the date of death with the HRV indices from each week, starting from 7 weeks to 1 week before death, with a repeated measures ANOVA. This analysis found that the ANN and RMSSD values decreased from 3 weeks before the date of death. Furthermore, for a more precise estimation of when the changepoint occurred, we conducted a piecewise regression analysis. This analysis revealed the changepoints for the indices in days before the patients’ deaths for ANN (–34.5, 95% CI –42.5 to –26.5 days), SDNN (–33.0, 95% CI –40.9 to –25.1 days), and RMSSD (–35.0, 95% CI –42.3 to –27.7 days). The results indicate that if the changepoint can be detected during BCG monitoring, it may be possible to predict the survival time from that changepoint: approximately 1 month later. Accurate estimation of the time of death approximately 1 month in advance by continuously monitoring HRV indices obtained from BCG could provide families or health care providers with more time to prepare for end-of-life care. Although our results are from a retrospective study and the sample size was small, to our knowledge, this is the first study to analyze data from long-term monitoring of older patients without cancer at end of life using HRV indices estimated from BCG.

HRV measures the time interval between adjacent heartbeats, that is, the variation in the time interval from R wave to R wave (ie, RRI) on the ECG. HRV indices are a useful noninvasive means of assessing autonomic function. The gold standard for evaluation of HRV indices is to evaluate the RRI in an ECG. However, in recent years, other systems have been developed to acquire BCG signals, most of which come in the form of a mattress or chair [34], and BCG is considered to be a potential substitute for HRV indices [35,36]. Martín-Yebra et al [36] evaluated the JJ, II, KK, and HH intervals of the BCG signal as an alternative to the ECG’s RRI and showed that the JJ interval was almost consistent with the RRI measured simultaneously with BCG. We also analyzed the HRV indices estimated from the JJ intervals of the BCG signal in our study.

The rapid aging of the world’s population and the widespread use of HRV have increased interest in the prognostic value of HRV in older patients, even outside the specific field of cardiology [37]. Kurita et al [38] examined the survival of patients based on HRV and blood tests such as serum albumin and C-reactive protein levels at admission among older Japanese individuals in special nursing homes. They reported that there was no statistically significant difference between the death and survival groups in terms of blood test values. However, the SDNN and coefficient of variables of RRI values, which have been suggested to be related to parasympathetic activity, were statistically higher in the survivor group. In another study, which used HRV to predict survival time for terminally ill patients with cancer [39], the length of survival time was shorter in groups with lower SDNN (21.3 ms or less) or higher heart rates (100 or more beats per minute) measured at baseline. Another prospective study using HRV to evaluate the prognostic values for discharge from the hospital in cancer patients compared the high-frequency (HF) and low-frequency components of HRV at admission between patients who were discharged and those who died. In the study, none of the HRV indices were statistically different, but the estimated value of the HF components of HRV as an index reflecting parasympathetic nerve activity (vagal activity), tended to be higher in the discharged group [40]. Since our study also showed a decrease in ANN, which represents an increase in heart rate, and a decrease in SDNN and RMSSD, which reflect parasympathetic function, it may be appropriate to use HRV indices related to parasympathetic function to make prognoses at end of life in older patients.

Our results showed a change in HRV indices approximately 35 days before patients’ deaths. A previous study of signs of mortality and the timing of their appearance in end-of-life patients in Japan [41] was based on the subjective perceptions of nurses working at a special nursing home for older people; it reported that the signs of mortality were divided into 19 categories and classified them according to the time they appeared. The study found that some signs appeared approximately 1 month before death and others appeared 2 days before. Signs that appeared 1 month prior to death included lack of eyesight, paleness, lack of vitality, somnolence, and decreased food intake compared to previous stable daily activities. Notably, the changepoint of the HRV indices was observed approximately 1 month before death in our study, and there may be some relationship between the signs recognized by the nurses and the change in HRV indices, that is, they may run in parallel. Therefore, in the future, it may be possible to construct a more accurate model that can predict the survival time of patients by combining more objective changes in HRV with the subjective symptoms identified by nurses.

### Strength

The strength of this study lies in the analysis of data collected continuously from patients for more than 2 months. For example, the Palliative Performance Scale (PPS) and Palliative Prognostic Index (PPI) scores at admission are commonly used as prognostic tools for terminal cancer patients [42]. However, many studies rely solely on values at admission to assess prognoses. In contrast, Kao et al [43] postulated that using only the PPI score on the first day may be limited because it does not consider subsequent changes in the patient’s condition. They showed that combining PPI scores from day 1 and 1 week later improved prognostic accuracy. In addition, Chan et al [44] examined changes in PPS scores at admission, week 1, and week 2 in terminally ill patients to predict patient survival. They found that all of these changes were independent predictors, with the greater the change, the higher the hazard ratio. These studies underscore the importance of analyzing changes over time. Although it is relatively convenient to measure the items needed to calculate PPS and PPI in a clinical setting, it is burdensome to observe these items daily. Therefore, if the sheet-type device used in this study can be used to measure HRV indices without inconvenience to the individual and facilitate making prognoses in end-of-life patients, it could be a very useful approach.

### Limitations

There are several limitations in this study. First, it was retrospective and used existing data from a single institution; therefore, selection bias was unavoidable. Second, the study used a small amount of data from 15 patients, which could have affected the statistical validity. Third, the patients’ detailed background information was not available, and the study included patients with a variety of underlying diseases. Therefore, the results of this study may have been influenced by chance or by confounding factors, such as the patients’ medical histories and medical conditions while they still survived.

### Conclusions

To our knowledge, this is the first study to assess HRV changepoints estimated from BCG signals in older patients at end of life. We found changepoints occurred approximately 1 month before a patient’s death, indicating that these changes could be used to predict patients’ deaths. This study also suggests the possibility of developing more objective and accurate predictive tools and offers valuable insights for future research. Such tools could be created by integrating BCG data with the subjective judgments of nurses, caregivers, and other health professionals, as well as established tools like the PPS, to make prognoses in cancer patients. However, since the findings were obtained in a retrospective study, future research is needed to determine how to detect these changes when observed prospectively.

## Abbreviations

ANN: average of normal-to-normal interval
BCG: ballistocardiography
ECG: electrocardiogram
HF: high frequency
HRV: heart rate variability
PPS: Palliative Performance Scale
PPI: Palliative Prognostic Index
RMSSD: root mean square of successive differences
RRI: RR interval
SDNN: SD of normal-to-normal interval

## Data Availability

The data sets generated during and/or analyzed during this study are not publicly available due to the confidential nature of the information, and consent to share data with outside parties has not been obtained from the patients.
